# Cytogenetic analysis of 3387 umbilical cord blood in pregnant women at high risk for chromosomal abnormalities

**DOI:** 10.1186/s13039-020-0469-6

**Published:** 2020-01-23

**Authors:** Yanmei Sun, Pingping Zhang, Ning Zhang, Limin Rong, Xiaoping Yu, Xianghua Huang, Yali Li

**Affiliations:** 1 Graduate school of Hebei Medical University, Shijiazhuang, 050017 P. R. China; 2 Department of Reproductive Genetic Family, Hebei General Hospital, Shijiazhuang, 050051 P. R. China; 3 Department of Obstetrics and Gynecology, the Second Hospital of Hebei Medical University, No. 215 West Heping Road, Shijiazhuang, 050000 P. R. China

**Keywords:** Cordocentesis, Prenatal diagnosis, Chromosome polymorphism, Mosaic, Cytogenetics, NIPT

## Abstract

**Background:**

Cordocentesis in our practice is most commonly indicated for rapid karyotyping in the second or third trimester and is regarded as the gold standard for foetal chromosomal aberration diagnosis in pregnancies at high risk for chromosomal abnormalities. In this study, we investigated 3387 umbilical cord blood samples for karyotyping from pregnant women who underwent cordocentesis and explored the pregnancy outcomes of foetal sex chromosome mosaicism and chromosomal polymorphism.

**Results:**

Out of the 3387 samples, 182 abnormal karyotypes were detected. Ultrasound soft markers were the most common prenatal diagnostic indication, but the detection rate of abnormal karyotypes was 2.02%, while it was 46.97% in the genome-wide NIPT-positive group. The rate of aneuploidy was lower in the soft marker group than in the other groups. Out of 16 cases with sex chromosome mosaicism, three pregnant women with foetuses with a lower proportion of sex chromosome mosaicism delivered healthy foetuses; the foetus with karyotype 46,X,i(Y)(q10)[20]/45,X[6] showed unclear genitals. Three foetuses with chromosomal polymorphisms had postnatal disorders.

**Conclusions:**

NIPT should not be recommended as the first-tier screening for chromosomal aberration for pregnancies with ultrasound soft markers or pathological ultrasound findings, but NIPT can be considered an acceptable alternative for pregnancies with contraindications to cordocentesis or the fear of procedure-related foetal loss. Mosaicism found in amniotic fluid cell culture requires further cordocentesis for karyotype confirmation, and the continuation of pregnancy is safe when a normal karyotype is identified in foetal blood culture. Further genetic testing and parental karyotype analysis are needed for foetal chromosomal polymorphisms.

## Introduction

Chromosome abnormalities may cause mental retardation, multiple dysplasia or malformation; however, until now, there have been no effective treatment measures. Therefore, it is very vital for chromosome diseases to be detected before birth. Ultrasound, maternal serum screening (MSS), and non-invasive prenatal DNA testing (NIPT) are commonly used screening methods for chromosomal aberrations at present; however, they are not suitable for accurate genetic testing. Amniocentesis or cordocentesis sampling for karyotype analysis is currently the gold standard for the prenatal diagnosis of foetal chromosomal diseases.

Cordocentesis is a prenatal diagnostic procedure for foetal blood sampling; it is well accepted as an efficient and relatively safe procedure for foetal genetic diagnosis, especially for karyotype confirmation after amniocentesis in the second or third trimester [1, 2]. Cordocentesis can also be used to diagnose foetal haemolytic disease, for intrauterine foetal blood transfusion and for intrauterine therapy [3]. As an invasive prenatal sampling procedure, cordocentesis inevitably carries a certain risk of pregnancy complications, such as paracentesis-related foetal loss and bleeding [4, 5]. Whether NIPT should replace cordocentesis for some pregnant women at high risk for foetal chromosomal abnormalities (e.g., abnormal MSS or ultrasound soft marker) has yet to be determined. In this study, 3387 samples of umbilical cord blood with different indications in the second or third trimester were obtained for karyotype analysis by ultrasound-guided cordocentesis. The rates of abnormal karyotypes were analysed among the different indications for prenatal genetic cordocentesis, and the mosaicism of sex chromosomes and chromosomal polymorphisms were noted for further follow-up.

## Methods

This was a retrospective study. A total of 3387 pregnant women at high risk for foetal chromosomal abnormality with gestational age ≥ 24 weeks underwent cordocentesis for rapid prenatal karyotyping in the Department of Reproductive Genetic Family of Hebei General Hospital between January 2011 and December 2018; failed sampling was excluded. All the pregnant women were enrolled in this study after written formal consent. This research was approved by the Ethics Committee of Hebei General Hospital. The indications for prenatal genetic cordocentesis were divided into the following groups: (i) NIPT positive; (ii) ultrasonographic structural abnormal findings (USAF); (iii) karyotype confirmation after amniocentesis (KCAA); (iv) ultrasound soft markers; (v) either mother or father with a chromosomal abnormality; (vi) advanced maternal age; and (vii) abnormal MSS.

Umbilical blood sampling was performed under sonographic guidance. Cord blood (2–2.5 ml) was collected for genetic testing. The traditional methods of single one-minute alkaline denaturation and foetal blood routine examination for mean corpuscular volume and mean corpuscular haemoglobin were performed to determine the purity of the sample. Cord blood cells were incubated in culture medium for 68–72 h at 37 °C, and then the cells were harvested and slides were prepared for chromosomal karyotype analysis according to the standard protocol. The prepared slides were placed on the GSL-120 high-throughput automatic chromosome scanning platform (Leica, Germany) for clone identification and scanning. G-band staining (plus C-band staining when necessary) was used for the chromosomal specimen preparation, and chromosomal analysis with targeted 320–400-band levels was performed. For each sample, 20–30 metaphases were counted, and 5 mitotic figures were analysed. When mosaicism or an abnormal karyotype was identified, the analysis was performed for a total of 50–100 mitotic phases depending on the number of cells that could be analysed. Fluorescence in situ hybridization (FISH) was performed when necessary according to the protocol of Dai et al. [6]. Chromosomal abnormalities were described in accordance with the International System for Human Cytogenomic Nomenclature 2016 (ISCN, 2016).

## Results

Out of 3387 samples obtained during invasive sampling procedures under ultrasound guidance, a total of 182 cases of abnormal karyotypes were detected, as well as 80 cases of chromosomal polymorphisms; the incidence of chromosomal abnormalities was 5.37%. Among all indications for prenatal genetic cordocentesis, ultrasound soft markers accounted for 73.16%; but the lowest rate of 2.02% was observed in the ultrasound soft markers group, while the rate was 11.17% in the USAF group and 19.30% in the advanced maternal age group. The rates of abnormal karyotypes in the NIPT-positive group were the highest (46.97%) (Table 1).

**Table 1:** Detection rates of abnormal karyotypes among different indications for prenatal genetic cordocentesis

Indication for genetic diagnosis	Cases (%)	Abnormal karyotype(n)	(%)
Ultrasound soft markers	2478 (73.16)	50	2.02
USAF	403 (11.90)	45	11.17
Advanced maternal age	114 (3.37)	22	19.30
Abnormal MSS	77 (2.27)	6	7.79
EMFCA	15 (0.44)	5	33.33
NIPT positive	66 (1.94)	31	46.97
KCAA	52 (1.54)	23	44.23
Total	3387	182	5.37

EMFCA: Either mother or father with chromosomal abnormality

USAF: Ultrasonographic structural abnormal findings

KCAA: Karyotype confirmation after amniocentesis

Of the 182 cases, 119 (65.38%) had chromosomal numerical abnormalities and 64 (35.16%) had chromosomal structural abnormalities; trisomy 21 was the most noted of the chromosomal numerical abnormalities, accounting for 32.42%, while chromosomal inversion was the most common of the chromosomal structural abnormalities, and 24 out of 26 cases were pericentric inversions on chromosome 9 (Table 2). There were 37 cases of sex chromosome abnormalities, 16 of which were sex chromosome mosaicism.

**Table 2:** Abnormal karyotype distribution

Abnormal karyotypes	Cases(n)	(%)
Numerical abnormalities	119	65.38
Trisomy 21	59	32.42
Trisomy 18	17	9.34
Trisomy 13	2	1.10
45,X	2	1.10
47,XXX	5	2.75
47,XXY	7	3.85
47,XYY	6	3.30
48,XXXX	1	0.55
Mosaic	19	10.44
Structural abnormalities	64	35.16
Translocation	15	8.24
Inversion	26	14.29
Derivative chromosome	7	3.85
Deletion	5	2.75
Duplication	1	0.55
Additio	5	2.75
Marker chromosome	3	1.65
Others	2	1.10
Total	182	100.00

Autosomal aneuploidy (trisomy 21, 18, and 13) mainly occurred in the NIPT-positive and USAF groups. Sex chromosomal abnormalities mainly occurred in the NIPT-positive group, while mosaics mainly occurred in the KCAA group. Chromosomal deletion, duplication, addition and derivative chromosome mainly occurred in the USAF group, while chromosomal translocation and inversion mainly occurred in the ultrasound soft marker group.

Of 403 cases in the USAF group, 45 cases of abnormal karyotypes were detected; therein, autosomal aneuploidy (trisomy 21 or 18) accounted for 46.67%, while in the ultrasound soft marker group, 50 abnormal karyotypes were detected out of 2478 cases, and autosomal aneuploidy (trisomy 21 or 18) accounted for 28% (Table 3). There were 52 cases that underwent genetic cordocentesis due to abnormal karyotypes in amniotic fluid cell culture, and 29 cases were verified to be normal karyotypes in foetal blood culture. The pregnant women continued pregnancy and delivered healthy neonates. Twenty-three cases were confirmed to be abnormal karyotypes in foetal blood karyotyping, of which 10 cases were mosaics (Table 3).

**Table 3:** Abnormal karyotypes in different indication for prenatal genetic diagnosis

Abnormal karyotypes	I	II	III	IV	V	VI	VII
Trisomy 21	18	10	3	12		13	3
Trisomy 18	3	11		2		1	
Trisomy 13	1					1	
45,X		2					
47,XXX	4		1				
47,XXY	1	1	1	2	1	1	
47,XYY	1		3	1		1	
48,XXXX	1						
Marker chromosome			3				
Mosaic	1	4	10	2	1	1	
Translocation		3	1	9	1	1	
Inversion		2	1	19	1	1	2
Derivative chromosome	1	5		1			
Deletion		1		2	1	1	
Duplication		1					
Additio		3				1	1
Others		2					

I, NIPT positive; II, Ultrasonographic structural abnormal findings (USAF); III, Karyotype confirmation after amniocentesis (KCAA); IV, Ultrasound soft markers; V, Either mother or father with chromosomal abnormality (EMFCA); VI, Advanced maternal age; VII, Abnormal MSS

There were 19 cases of mosaicism diagnosed prenatally in cord blood culture, involving 16 cases of sex chromosome mosaicism, as listed in Table 4. Further follow-up showed that 13 pregnant women chose to terminate their pregnancies, in which a foetus with karyotype 46,X,i(Y)(q10)[20]/45,X[6] showed unclear genitals. Three pregnant women elected to continue pregnancy with foetuses with karyotypes 45,X[3]/46,XX[97], 45,X[4]/46,XX[65], or 45,X[2]/46,XX[36] and delivered healthy neonates.

**Table 4:** Distribution of sex chromosome mosaicism

Karyotypes	Indications for cordocentesis	Pregnancy outcomes
45,X[12]/46,XX[8]	foetal growth retardation	Induced abortion
45,X[23]/46,XX[67]	NIPT abnormalities	Induced abortion
45,X[4]/46,XX[92]	Foetal atrial septal aneurysm	Induced abortion
45,X[11]/46,XX[44]	Karyotype confirmation after amniocentesis	Induced abortion
45,X[3]/46,XX[97]	Advanced maternal age	deliver a girl by caesarean section
45,X[4]/46,XX[65]	Karyotype confirmation after amniocentesis	deliver a girl by caesarean section
45,X[2]/46,XX[36]	Karyotype confirmation after amniocentesis	deliver a girl by caesarean section
45,X[30]/46,XX[50]	Karyotype confirmation after amniocentesis	Induced abortion
46,X,+mar[81]/45,X[17]/47,X,+2mar[2]	Karyotype confirmation after amniocentesis	Induced abortion
46,X,i(X)(q10)[28]/45,X[2]	Karyotype confirmation after amniocentesis	Induced abortion
46,X,del(X)(p11)[17]/45,X[10]/46,X,+mar[6]	Advanced maternal age	Induced abortion
45,X[21]/46,X,i(X)(q10)[20]/46,XX[59]	cisterna magna> 10 mm	Induced abortion
46,X,i(Y)(q10)[20]/45,X[6]	Karyotype confirmation after amniocentesis	Induced labour a boy without clear genitals
47,XXX[13]/46,XX[47]	Karyotype confirmation after amniocentesis	Induced abortion
47,XXY[10]/46,XY[42]	Foetal hydronephrosis	Induced abortion
47,XYY[48]/48,XXYY[20]	Karyotype confirmation after amniocentesis	Induced abortion

Out of 80 cases of chromosome polymorphism, 32 cases occurred on the Y chromosome, and the karyotype 46,XY (Y ≥ 18) accounted for 26.3%. Six pregnant women chose pregnancy termination due to pathological ultrasound findings. Among the 51 cases with the continuation of the pregnancy, one foetus with karyotype 46,XY (Y ≥ 18) died of postnatal pneumonia complicated by encephalitis, a foetus with karyotype 46,XX,15pstk+ suffered severe neonatal asphyxia after birth, and one foetus with karyotype 46,XY,15 ps- had mild varus pedis (Table 5).

**Table 5:** Chromosomal polymorphism in this study

Karyotypes	Cases(%)	Follow-up(n)		
		Loss to follow-up	Terminated gestation	Postnatal follow-up
46,Xn,1qh+	4 (5.00)	0	1	3
46,Xn,9qh+	9 (11.25)	0	2	7
46,Xn,13cenh−/cenh+/p+/ps−/pstk+	6 (7.50)	0	0	6
46,Xn,14pstk+	1 (1.25)	0	0	1
46,Xn,15pstk+/ps−/cenh+	10 (12.50)	2	1	7 (1 case of postnatal severe neonatal asphyxia, 1 case of postnatal mild varus pedis)
46,Xn,16qh+	3 (3.75)	1	0	2
46,Xn,21pstk+/pss	7 (8.75)	1	1	5
46,Xn,22pstk+/ps-	2 (2.50)	0	0	2
46,X,Yqh−/Yqs	6 (7.50)	1	0	5
46,XY(Y ≥ 18)	21 (26.25)	7	1	13 (1 case died of postnatal pneumonia complicated with encephalitis)
46,XY(Y ≤ 21)	11 (13.75)	1	0	10
Total	80	13	6	61

## Discussion

Cordocentesis sampling in our practice was used for rapid karyotyping, which is regarded as the gold standard for foetal chromosomal aberration diagnosis [2]. A total of 3387 pregnant women with different indications underwent genetic cordocentesis, and 182 abnormal karyotypes were detected; chromosome aneuploidy was the most common karyotype, accounting for 54.95%. Cordocentesis is associated with a risk of complication; the foetal loss rate within 2 weeks in the study was 0.47%, lower than that reported in other studies, in which cordocentesis was performed in pregnancies with a gestational age of < 24 weeks (early cordocentesis is more difficult and has a tendency to increase the rates of adverse outcomes) [7].

Ultrasound soft markers are defined as minor nonspecific findings on ultrasound scans, which are often transient and have little or no pathological significance, such as an echogenic intracardiac focus, a thickened nuchal fold and an echogenic bowel; although not pathological per se, ultrasound soft markers have been found to be linked to foetal aneuploidies such as Down syndrome [8]. In this study, the most common indication for diagnostic genetic testing was ultrasound soft markers, which accounted for 73.16% of the indications, and the rate of foetal chromosome abnormality was 2.02% (50/2478). Whether other techniques can be used as first-tier tests to detect chromosomal abnormalities in pregnancies with ultrasound soft markers remains unknown. The introduction of NIPT offered an alternative for genetic evaluation with high sensitivity and specificity [9, 10]. Our study showed that trisomy 21, 13 or 18 was identified in 14 of the 50 cases of abnormal karyotypes detected in the ultrasound soft marker group. If NIPT had been used as the first-tier screening for chromosomal aberration, it would have missed 26/50 abnormal karyotype diagnoses, indicating that NIPT cannot replace the invasive diagnostic genetic procedure due to false negatives in pregnancies with ultrasound soft markers or abnormal ultrasound findings, which was consistent with the findings of a previous study [11]. NIPT may be considered an acceptable alternative for pregnancies with invasive prenatal diagnosis contraindications or the fear of procedure-related foetal loss.

The positive predictive value of NIPT in the study was 46.97% because the NIPT applied in the study was a genome-wide non-invasive foetal aneuploidy detection method that was not limited to the rapid diagnosis of chromosomes 13, 18, and 21 and the sex chromosomes. There were 18 cases of Down syndrome detected in the study, with 1 false-positive result, which is similar to the results of a previous study [12].

In this study, 29 cases of abnormal karyotypes found in amniotic fluid cell culture were identified as normal karyotypes in foetal blood culture; this was probably because the sources of amniotic fluid cells in the second trimester were often diversified, and amniotic fluid cells can be mixed with cells derived from the placenta or maternal cells; moreover, poor growth cultures of amniotic fluid cells can induce pseudomosaicism [13]. Diagnostic cordocentesis for karyotype analysis was valuable for abnormal karyotype confirmation in amniotic fluid cell culture [14]; the continuation of the pregnancy was safe when a normal karyotype was detected in foetal blood culture.

Further follow-up for the pregnancy outcomes showed that most pregnant women with mosaicism chose to terminate the pregnancy, and 3 pregnant women continued the pregnancy and delivered healthy neonates, indicating that sex chromosome mosaicism should not be taken as the indication for pregnancy termination. A foetus with karyotype 46,X,i(Y)(q10)[20]/45,X[6] had unclear genitals. Sex chromosomes tend to incur non-disjunction and recombination mistakes because of their special structure and gene content compared with autosomes. The phenotype of sex chromosome mosaicism can be inconsistent with the mosaic proportion, which may be related to abnormal X inactivation in the early stages of embryogenesis. However, research on this question has not been carried out in this study due to sample limitations. Further studies are needed to expand the sample size.

Chromosomal polymorphism, also known as chromosomal normal variation, is defined as the constant small variations in chromosome morphology, including variation in length, number and position, and is usually without obvious manifestation and pathological significance. Chromosomal polymorphism has been reported to be associated with some disorders, such as recurrent miscarriage, stillbirth, foetal death, azoospermia, and infertility [15–17]. In this study, 80 cases of chromosomal polymorphism were detected, while inversion variants were classified as structural chromosome rearrangements according to ISCN 2016. Three cases of polymorphism incurred postnatal conditions such as severe neonatal asphyxia and mild varus pedis, but the clinical features cannot reliably be linked to the karyotype due to a lack of deep sequencing. Further genetic testing and parental karyotype analysis were needed for foetal chromosome polymorphisms.

## Conclusion

Pregnant women at high risk for foetal chromosomal abnormality should be offered further prenatal genetic testing. NIPT cannot replace the invasive diagnostic genetic procedure and may be considered an acceptable alternative for pregnancies with invasive prenatal diagnosis contraindications or the fear of procedure-related foetal loss. Mosaicism found in amniotic fluid cell culture requires further cordocentesis for karyotype confirmation, and the continuation of the pregnancy is safe when a normal karyotype is identified in foetal blood culture; the prognosis is good for foetuses with a lower proportion sex chromosome mosaicism. Further genetic testing and parental karyotype analysis were needed for foetal chromosomal polymorphisms.

## Data Availability

All data generated or analyzed during this study are included in the article.

## Abbreviations

NIPT: non-invasive prenatal DNA testing; FISH: fluorescence in situ hybridization
ISCN: International System for Human Cytogenomic Nomenclature
KCAA: karyotype confirmation after amniocentesis
MSS: maternal serum screening
USAF: ultrasonographic structural abnormal findings
